# The ripple effects of funding on researchers and output

**DOI:** 10.1126/sciadv.abb7348

**Published:** 2022-04-22

**Authors:** Reza Sattari, Jung Bae, Enrico Berkes, Bruce A. Weinberg

**Affiliations:** 1 Canada Mortgage and Housing Corporation, Ottawa, Canada.; 2 Ohio State University, Columbus, OH 43210, USA.; 3 National Bureau of Economic Research (NBER), Cambridge, MA 02138, USA.; 4 IZA Institute for the Study of Labor, Bonn 53113, Germany.

## Abstract

Using unique, new, matched UMETRICS data on people employed on research projects and Author-ity data on biomedical publications, this paper shows that National Institutes of Health funding stimulates research by supporting the teams that conduct it. While faculty—both principal investigators (PIs) and other faculty—and their productivity are heavily affected by funding, so are trainees and staff. The largest effects of funding on research output are ripple effects on publications that do not include PIs. While funders focus on research output from projects, they would be well advised to consider how funding ripples through the wide range of people, including trainees and staff, employed on projects.

## Teaser

NIH funding stimulates research by generating ripple effects through trainees, staff, and others employed on projects.

## INTRODUCTION

The scientific community’s understanding of how science is produced is remarkably limited. When trying to understand the production of science, funders have often focused on the effects of grants, including funding amounts and mechanisms. However, funding and grants do not produce science directly (*1*–*5*). While biographers, popular writers, and psychologists have realized the critical role played by people in the production of science, they have tended to focus on a small number of eminent individuals (e.g., Albert Einstein) and delved into their life experiences (*6*–*7*), although it has been widely recognized that science is increasingly produced by teams (*8*). Lacking large-scale, quantitative data, researchers have often turned to ethnographies and case studies to understand the role of teams and communities in science (*9*–*10*). Even scientific rhetorical conventions—the use of the passive voice, the “royal we,” and the focus on formal hypothesis testing, methods, and findings rather than the research process itself—serve to obscure rather than illuminate how research is actually produced.

In this paper, we peer inside the black box of science production and find that funding for “science” primarily supports the scientists, including trainees and staff, who produce it. Turning our focus from projects (or great innovators) to teams raises a completely new set of questions: Who are the people that produce research in terms of gender, race, ethnicity, and age? What roles do they play in labs? In addition, given the importance of trainees, how does the support from research projects ripple through to their future productivity?

We are able to answer these questions thanks to a new match between two unique datasets. First, the UMETRICS data provide record-level information on payments on sponsored research projects at 72 university campuses comprising 41% of academic R&D in the United States (*11*–*13*). Starting from transactions on projects, we use payments to identify all people working on research projects—from faculty members to trainees and staff—regardless of how many (if any) articles they appear in as authors. We then match publications to research projects in a novel way by identifying the articles written by the people who work on those research projects, whether or not the articles acknowledge the project itself or are even conducted as part of the project. Prior work has often relied on grant acknowledgements in publications (*1*–*3*), which are sometimes misreported, and ignored people who have been working on a project but are not named as authors. Moreover, our person-based approach allows us to cast a much wider net and consider, for example, trainees who are supported on a research project but also do important work separate from that project, perhaps after leaving the lab. The conventional, acknowledgement-based approach to identifying publications has no way of capturing these indirect “ripple effects,” which means that it misses a potentially large portion of the effects of funding. By contrast, our approach begins with the people who conduct research and their publication trajectories, and it therefore captures these ripple effects.

UMETRICS allows us to identify who is touched by research funding and what they do. It contains data on job titles, which allow us to describe the mix of positions supported, and names, from which we impute individual characteristics, such as gender, race, and ethnicity.

Our second core dataset is the Author-ity disambiguation of PubMed (*14*–*15*), which permits us to identify the lifetime publications of the researchers in UMETRICS. Author-ity algorithmically identifies all the articles authored by unique individuals in PubMed. These publications are matched to employees in UMETRICS using, first, the grant acknowledgements in National Institutes of Health’s (NIH’s) ExPORTER from 1985 through 04 February 2020 and, second, a novel, network-enhanced, fuzzy name–matching approach detailed in Material and Methods.

We also use data on funding from NIH’s ExPORTER. All funding amounts are expressed in 2018 dollars using the Biomedical Research and Development Price Index (BRDPI). Our funding and output data are aggregated to the level of lab years or principal investigator (PI) years, defined as all the projects with the same PI in a given year. (Note that, in this paper, “lab” is used to refer to a group that is run by a certain PI, and the two concepts are equivalent in terms of our unit of analysis.) We estimate all models in levels and afterward obtain implied percentage changes by dividing estimates by means. Compared to a logarithmic model, this approach ensures that large labs influence the estimates substantially. Publications are assigned to all labs or PIs that supported one or more authors on at least one project. Summary statistics are reported in Materials and Methods.

## RESULTS

### What does science funding support?

**Fig. 1.**
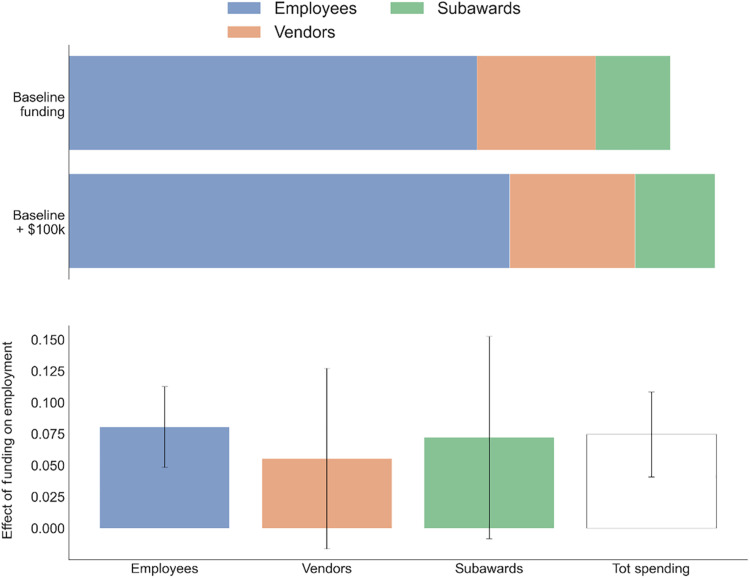
Allocation of science funding. (**Top**) It shows how (direct) spending is allocated across employees, vendors, and subawards for a lab of the mean size of $362,198 in 2018 BRDPI dollars (baseline) and a lab with $100,000 in additional funding. (**Bottom**) Percentage change in spending in each category when the lab experiences a $100,000 increase in total funding (calculated using the changes in spending in each category from a $100,000 increase in funding divided by the initial spending on that category) along with 95% confidence intervals. SEs are clustered at the lab level. These are estimated from a regression of spending in each category on total funding for that lab in each year controlling for lab or PI FEs, calendar year FEs, and for the number of years since a PI first received NIH funding. We divide the estimate on funding by the mean spending in each category across all lab years. Tot spending, total spending.

We begin our empirical investigation with an analysis of how science funding is allocated across very broad categories of activity. We divide direct costs into three components: employees of all types, subawards to other institutions, and spending on purchased inputs from vendors, such as materials, supplies, and travel. The top of Fig. 1 shows how direct spending is allocated across these spending categories for a lab of the mean size of $362,198 in 2018 BRDPI dollars (baseline) and a lab with $100,000 in additional spending. Spending on employees accounts for over two-thirds (68%) of total spending, followed by purchases from vendors and subawards. When the funding for a lab increases by $100,000, three-quarters of the direct spending is allocated to employees. Assuming that individual workers are paid according to their productivity (and that wages are not highly responsive to funding amounts), the estimates in Fig. 1 can be interpreted as measuring efficiency units of labor (i.e., the effect on labor inputs weighted by wages). Almost half of the remainder gets allocated to subawards, much of which likely funds staff at the subaward recipient’s lab. The bottom shows the percentage change in spending in each category from a $100,000 increase in funding (along with 95% confidence intervals), with employee spending having the largest percentage response, followed by subawards.

Biomedical research spans many substantive areas. The NIH is divided into 27 institutes and centers (ICs), most of which are focused on a specific organ or disease. To analyze topical differences here and below, we generate estimates for the ICs for which we have sufficient data. While the overwhelming majority of projects sponsored by different ICs allocate the largest portion of their funds to employees, fig. S1A shows some differences across ICs. For instance, a greater share of awards from the National Institute on Alcohol Abuse and Addiction (NIAAA) and the National Institute of Environmental Health Sciences are allocated to vendor purchases.

**Fig. 2.**
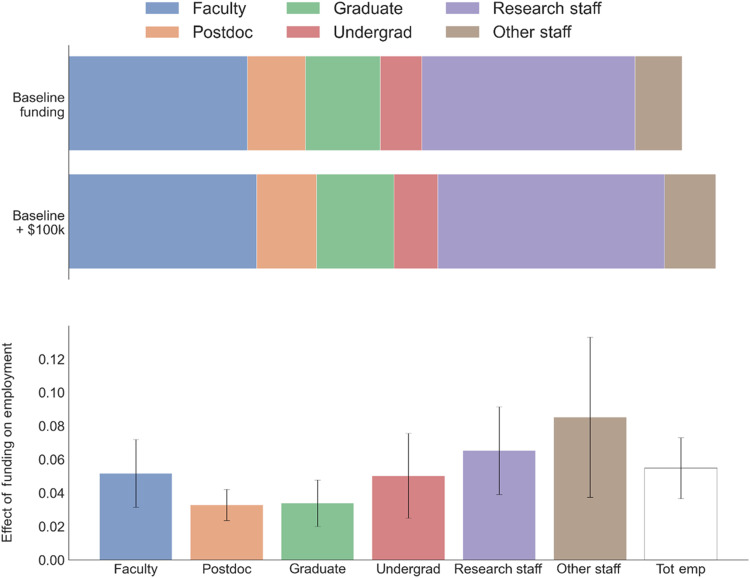
Allocation of science spending across occupations. (**Top**) It shows how (direct) spending is related to the employment measured in full-time equivalent workers for different types of employees for a lab of the mean size of $362,198 in 2018 BRDPI dollars (baseline) and a lab with $100,000 in additional spending. Full-time equivalent workers are estimated by prorating people by the share of their time charged to each project, their full-time/part-time status, and the number of days worked during the year. (**Bottom**) Percentage change in employment measured in full-time equivalent workers in each category from a $100,000 increase in funding (along with 95% confidence intervals). SEs are clustered at the lab level. These are estimated from a regression of employment in each category on total funding for that lab in each year controlling for lab or PI FEs, calendar year FEs, and for the number of years since a PI first received NIH funding. We divide the estimate on funding by the mean spending in each category across all lab years. Tot emp, total employment.

Given the primary role played by personnel, we next turn to study what types of personnel are employed on projects. UMETRICS provides data on each person employed, including the amount of time allocated to individual projects by pay period (typically a month). We observe employee job titles, which have been aggregated into six major categories, namely, faculty, postdocs, graduate students, undergraduate students, research staff, and other staff (this last category includes people who were not classified elsewhere, who are overwhelmingly staff based on manual inspection). Research staff includes people categorized as research and research facilitation, while other staff includes all the employees that are categorized as technical support, clinical, instructional, and other. Table S1 reports the breakdown of occupations before the aggregation. Here too, the unit of observation is a lab or PI year. Figure 2 shows the effect of funding on employment measured in full time equivalent workers by job categories. The employment of research staff and faculty, not all of whom are PIs, responds the most to funding increases, although, in percentage terms, other staff is even more responsive. Undergraduates are also highly responsive in percentage terms, but they have the lowest base of all categories. These findings indicate that larger labs are more professionalized than smaller labs, with larger shares of faculty and staff.

Figure S1B provides employment breakdowns by NIH ICs. Again, while there is a high degree of similarity across ICs, there are some interesting and plausible patterns. For instance, a larger share of employees supported by the National Institute of General Medical Sciences are trainees (graduate students or postdocs), and projects funded by the National Center for Advancing Translational Sciences support considerably more personnel (over 80) than any other IC.

### What does science funding produce?

The goal of biomedical research is to produce knowledge, particularly knowledge that leads to improvements in health. We therefore turn to study the research output from projects. Because the most productive researchers are likely to obtain the most funding, simply relating publications to funding would confound the causal effect of funding on output with underlying unobserved factors that attract funding and are associated with higher output (*16*–*21*). To tease out causal effects in a widely accepted and intuitive way, we rely on an “event study” design. Here, we relate research output to leads (i.e., future values) and lags (i.e., past values) of funding. Intuitively, if there is a causal effect of funding on output, then one would expect to observe an increase in publications in the years after funding is received but not in the preceding years. In other words, future funding amounts should not affect past output and are included in the regression model as a diagnostic to verify whether the model successfully controls for unobserved factors that might confound the estimated relationship between funding and productivity. If future funding is related to current output, that would be an indication that the model is likely misspecified and does not adequately control for the unobserved factors relating output to funding. Frequently, but not always, event study designs are applied to analyze the effect of a discrete change in a policy at a point in time on future outcomes. By contrast, we study changes in the amount of funding, so the estimates can be interpreted as the effect of increasing funding by $100,000 in a given year on publications in surrounding years.

We focus our analysis on fixed effect (FE) regression models that include FEs (i.e., indicator/dummy variables) for each PI or lab that sweep out all time-invariant differences across PIs, including differences stemming from differences across fields. The FEs are expected to control for unobserved determinants of productivity and funding. In the Supplementary Materials, we also estimate ordinary least squares (OLS) models that do not account for these FEs to explore how our estimates change when we do not control for unobserved factors. If our intuition holds, we expect to observe future funding being related to past publications in the OLS models but not in the FE models. This would suggest that the noncausal portion of the relationship between funding and output may be adequately captured by accounting for fixed differences across PIs. Both models also include dummy variables for calendar years and for the number of years since a PI first received NIH funding, a proxy for experience, in addition to leads and lags in funding.

**Fig. 3.**
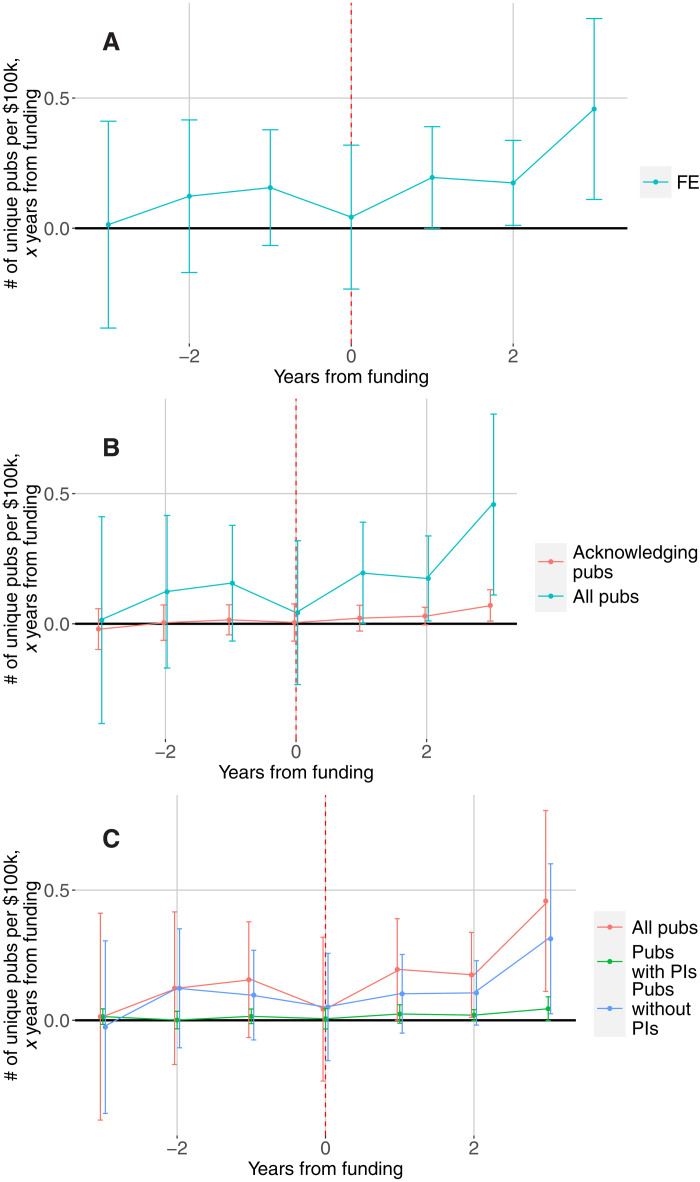
Event study analysis of funding and publications. (**A**) Publications authored by people on awards, FE estimates. (**B**) Publications authored by people on awards versus those that also acknowledge awards. (**C**) Publications with and without the PI as author. Note: The figure plots the relationship between funding to a lab (measured in 2018 BRDPI dollars) and unique publications authored by the people in that lab, from publications 3 years before funding is received (−3) to 3 years after funding was received (+3). (A) Estimates for all unique publications using lab or PI fixed effects (FE) models, which account for all time-invariant differences across labs. (B) FE estimates for all unique publications authored by people in that lab and the subset of those publications that also acknowledge the awards that fund that lab. (C) FE estimates for all unique publications authored by people in that lab, those including the PI as an author (with or without other lab personnel), and those without the PI as an author. In some cases, we are not able to determine whether the PI is an author on a publication, so the latter two series do not sum exactly to the former. All estimates control for calendar year FEs and for the number of years since a PI first received NIH funding. Ninety-five percent confidence intervals are shown in all panels. SEs are clustered at the lab level.

Figure 3A plots the results from a basic event study design where the outcome is the number of unique publications in a focal year by personnel ever observed employed on a PI’s grants. The independent variable of interest is total NIH funding in the 7 years around the focal year (the 3 years before the focal year, the focal year, and the 3 years after the focal year). The FE estimates show more publications for labs with higher funding in the past. The estimates indicate roughly 0.8 more publications for each additional $100,000 in funding, with over half of that effect coming in the third year after funding. Reassuringly, in this case, the estimates show a weak relationship between past publications and future funding, suggesting that the PI FEs adequately control for unobserved differences in publication records across researchers that affect funding decisions. Despite differences in time periods, funding sources, and units of analysis, the magnitudes are broadly similar to existing causal estimates (*16*, *21*), which estimate an increase of about 10 publications per $1 million in additional funding.

To illustrate the consequences of inadequately controlling for productivity, we report OLS estimates in fig. S2A (along with the FE estimates). The OLS estimates indicate that publications are strongly related to funding received in the years after the publications but that past funding is weakly related to future publications. These results suggest that funding is allocated to labs or PIs with stronger publication histories and point to the importance of including PI FEs.

Although the absence of significant effects before funding suggests that the FE approach appropriately controls for pretrends and differences in productivity across labs and fields, there are unobserved factors that might confound the interpretation of our results. In particular, two unobserved factors might be at play, both of which would likely bias our estimates upward. First, funding might allow labs to hire researchers who are, on average, more productive (of course, rapid lab expansion could also lead to a reduction in the ability of hires). Second, on the basis of the content of grant applications, for instance, the NIH may be able to identify increases in future lab productivity in advance and channel resources to the labs whose productivity is about to increase. Our FE event study design reduces the risk of differences in lab productivity, in that the FEs eliminate all time-invariant differences in productivity across labs and the event study pretrends allow us to identify whether productivity increases before receiving funding. At the same time, without (quasi-) experimental variation, we cannot eliminate these possibilities. It is also worth noting that our FE estimates of the direct effects (i.e., abstracting from ripple effects) are in line with those reported in papers that use different identification strategies, which is somewhat reassuring in terms of the validity of our empirical approach.

Our main estimates focus on all the articles written by people who are employed on a PI’s grants, while most research has focused on publications that acknowledge grants (*1*–*3*). There are a number of reasons why these two approaches might diverge. One is simple reporting error—not all publications properly acknowledge the grants that support them. Historically, there was under-acknowledgement of awards, which might be another explanation for the large ripple effects, but due to changes in NIH policies, under-acknowledgement of awards is now likely greatly reduced. Today, it is more likely that some publications acknowledge grants to which they are only loosely related. Here, because we focus on all publications by people touched by funding, our outcome variable includes publications that are not directly connected to the PI’s grants but are produced by the people employed on those grants and who may benefit from an indirect “ripple effect.” Figure 3B repeats the FE estimates from Fig. 3A and adds FE estimates when the dependent variable captures all the publications that do acknowledge a PI’s award and are authored by one or more employees, including the PI her/himself. These estimates show a broadly similar pattern but are far lower than the estimates we obtained for the larger set of publications—roughly 0.12 articles per $100,000. This difference suggests very large ripple effects through people supported on grants, which are not captured in analyses that only consider publications acknowledging grants.

One way of assessing the role of ripple effects is to look at which personnel are listed on publications. We start from the assumption that publications that involve the PI are most likely directly related to a grant, while those without the PI are less likely to be directly supported by the grant. Figure 3C shows that the effects of funding on publications that include the PI as author are substantially lower and, interestingly, much closer to the ones found by (*18*), who consider PIs as their unit of analysis. These estimates suggest that the ripple effects of funding are quite large, that underreporting likely does not account for the gap between publications of people attached to grants and publications that acknowledge awards (in panel B), and that even if one is interested solely in the research outputs of grants, it is essential to study all the people that are supported on projects and their subsequent publications even when not directly related to the project itself.

Because funding leads labs to expand, one possible explanation for the increase in publications is that increases in funding simply increase the number of people who are connected to a PI whose publications are then counted. To address this concern, fig. S2B shows that our estimates are robust to controlling for the number of employees in the lab.

Given that the ultimate goal of biomedical research is to inform clinical practices and improve health, fig. S2C focuses on “clinical” publications, defined as clinical trials, systematic reviews, and clinical guidelines and also on “research” publications, as defined by the iCite database from NIH’s Office of Portfolio Analysis. A limitation of this measure is that only a small share of publications are clinical by this definition (2.1 per lab per year versus 27 publications of all types per lab per year). Therefore, as expected, the estimates for clinical publications are considerably smaller than the overall estimates. Nevertheless, they show a similar pattern, indicating that the effect of funding on clinical work is similar to the overall impact. In percentage terms, the effects on clinical publications are slightly smaller, with an elasticity of 0.025 for clinical publications versus 0.03 overall. On the other hand, given that most publications are research publications, the estimates for research publications are quite close to the estimates for all publications (e.g., in Fig. 3A).

Since funding increases the quantity of publications, it is natural to explore whether funding is associated with an increase or decrease in the quality of research. Figure S2D explores this question using the mean of NIH’s Relative Citation Ratio (RCR) and the maximum RCR of all the papers published by a given lab in a given year (*22*). It shows that the mean RCR is essentially flat in funding. The RCR of the top paper is higher in labs that receive more funding, but this relationship holds for funding both before and after the publication, suggesting that labs that are producing higher RCR work receive more funding but that funding does not increase the maximum RCR. Overall, funding does not appear to have a large effect on the quality of research. Our results are again broadly consistent with existing causal estimates (*16*, *21*), although they consider forward citations per publication.

The impact of funding may vary across funding mechanism and/or research area. Figure S2E provides estimates by funding mechanism—R01s only and all research grants and research program grants including R01s. The estimates for all research grants are similar to those for all funding, but the estimates for R01s turn out to be quite noisy. This is due to the reduction in sample size and variation in funding amounts.

We also explore possible heterogeneity of these results by institutional characteristics and career age of the PIs. Figure S2 (F to H) shows that our results are mainly driven by institutions in the top quartile in terms of the number of doctoral degrees awarded, enrollment, and federal funding. Furthermore, although the estimates are somewhat noisier, fig. S2I suggests that PIs with more experience benefit more from an increase in funding.

In understanding ripple effects, some publications occur after a grant ends, and/or people move to different projects. To quantify these effects, table S2 reports the distribution of papers by occupation and timing of publication relative to the employment in a specific lab. More precisely, we report the number of papers that were published before a researcher is paid by a certain lab for the first time in our sample, after the last transaction in our records, and between the two times. Note that we already exclude from our sample any paper published more than 1 year before a certain PI (or her institution) appears in UMETRICS. The plurality of papers (43.5%) were published while working in a specific lab, almost one fifth (19.3%) before the first payment and 37.3% after the last payment. Moreover, the rates vary in an intuitive way across occupations. For faculty, who are more closely attached to labs and likely to have flatter productivity profiles, only 32.5% of publications are after the last payment. By contrast, for trainees, who are expected to have steeper productivity profiles and less attachment to labs, 52.7% of postdoc publications, 60.2% of graduate student publications, and 65.0% of undergraduate publications are after leaving labs.

Again, to get at topical differences, we generate estimates for the NIH ICs for which we have sufficient data. For these estimates, we assign each PI to the IC that provides the most funding for her/his research in each year funded by NIH and then estimate the effect of all funding for each group of PIs. Note that although, in principle, the primary funder can change between years for a given PI, we have gone back to 2001 and found that the primary funder never changes for 90% of PIs over the course of their observed careers. The results, reported in fig. S3, tend to be clustered around the overall estimate (represented by the dashed line) and noisy. The National Eye Institute, National Institute on Aging (NIA), NIAAA, and the National Institute of Allergy and Infectious Diseases all have estimates that are statistically below the mean, while only the National Institute of Arthritis Musculoskeletal and Skin Diseases is statistically above the overall mean.

### Who does science funding support?

**Fig. 4.**
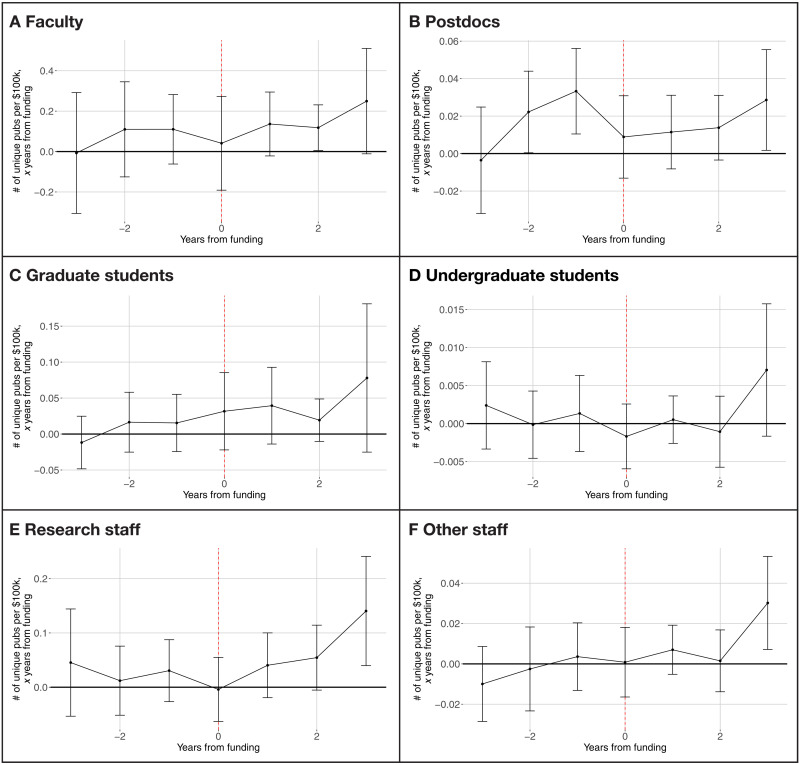
Event study analysis of funding and publications by occupation of author. (**A**) Faculty. (**B**) Postdocs. (**C**) Graduate Students. (**D**) Undergraduate Students. (**E**) Research Staff. (**F**) Other Staff. Note: The figure plots the relationship between funding to a lab (measured in 2018 BRDPI dollars) and unique publications authored by the people in that lab, from publications 3 years before funding is received (−3) to 3 years after funding was received (+3). Estimates are broken down by occupation of the author. An article is counted once (and only once) for each type of employee who is an author [i.e., if two authors are faculty and one is a postdoc, it will be counted once in (A) and once in (B)]. All estimates control for lab or PI FEs, which account for all time-invariant differences across labs., calendar year FEs, and for the number of years since a PI first received NIH funding. Ninety-five percent confidence intervals are shown on all figures. SEs are clustered at the lab level.

If, as we have shown, a full understanding of the role of funding requires an understanding of the people supported and their output, then it is interesting to study the characteristics of the people that publish as a result of increased funding. Figure 4 uses UMETRICS data on job titles to identify the effects on publications across roles and show how publications change for different types of people. Unfortunately, when the estimates are disaggregated by occupation, they become considerably noisier, especially for smaller groups (on the other hand, research staff are a relatively large group and experience a relatively smaller increase in SEs) and show pretrends for some occupations and therefore need to be interpreted with caution. The disaggregated point estimates suggest an increase in publications for all categories of employees, which is greatest in the last year, although the imprecision of the estimates makes it hard to draw firm conclusions or say whether the process is building gradually over time or is more discontinuous in year 3.

If one is willing to interpret magnitudes for imprecise estimates, the differences across groups are interesting. Specifically, the largest effects are for faculty (0.25 additional publications per $100,000), but most of the effect is among faculty other than the PI (as can be seen by comparing Fig. 4A for all faculty and the series for publications including PIs in 3C). However, given that the mean number of publications by faculty is 20.1, the percentage effect on faculty is more modest (1.2%). The effects on publications by graduate students, research staff, and nonresearch staff taken together are comparable to those for faculty, and, given that their baseline publication rates are considerably lower (2.1, 4.2, and 0.80, respectively), they all experience percentage effects around 3 to 4% per $100,000. Even undergraduates experience a ripple effect, albeit a relatively modest one. It is interesting to note that Fig. 4B suggests that postdocs also experience an increase in publications the year before their lab receives a grant. This increase might be due to anticipatory effects with PIs scaling up the lab before a grant becomes active. If correct, then such an anticipatory effect suggests that PIs have the ability to use money from one project to hire people for another project. If so, this is evidence that it is important to define labs using PIs instead of awards. Thus, the estimates at least suggest that the ripple effects from funding potentially spread across different types of employees, including faculty, with large percentage effects on staff and trainees.

The effects that we observe for graduate students and undergraduates might be driven by mobility. For example, it might be the case that graduate students obtain faculty positions after a couple of years working for a lab that receives more funding and start their own labs publishing more than otherwise. By contrast, students in underfunded labs might get discouraged and leave academia and therefore publish less than their peers. While table S2 suggests that these mechanisms are unlikely to be the primary drivers of our estimates, to the extent that these mechanisms operate, mobility might be considered part of the ripple effect if people working in a lab that experienced an increase in funding tend to move to more productive teams.

**Fig. 5.**
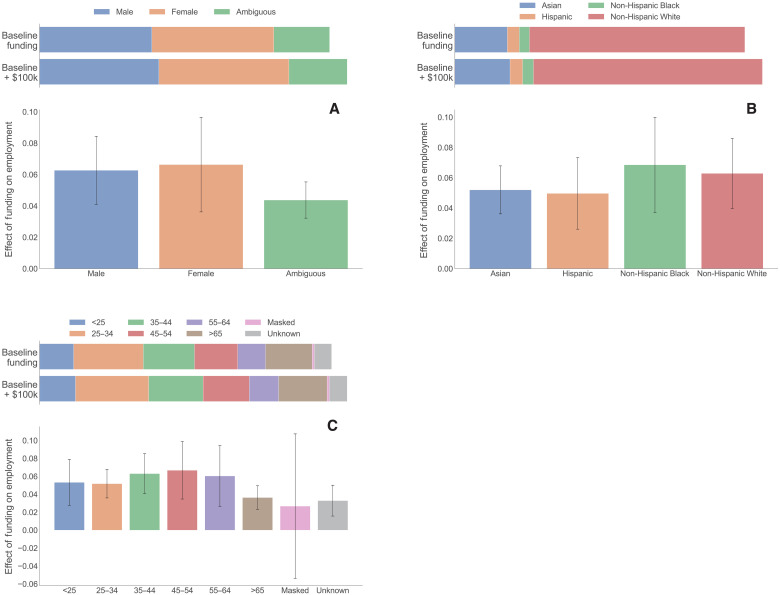
Demographic composition of projects. (**A**) Gender. (**B**) Race and ethnicity. (**C**) Age. Note: The top portion of each panel shows how (direct) spending is related to the employment measured in full-time equivalent workers with different characteristics for a lab of the mean size of $362,198 in 2018 BRDPI dollars (baseline) and a lab with $100,000 in additional spending. Full-time equivalent workers are estimated by prorating people by the share of their time charged to each project, their full-time/part-time status, and the number of days worked during the year. The bottom portion of each panel shows the percentage change in full-time equivalent workers of each type of employee from a $100,000 increase in funding (along with 95% confidence intervals). SEs are clustered at the lab level. These are estimated from a regression of employment of each type of employee on total funding for that lab in each year controlling for lab or PI FEs, calendar year FEs, and for the number of years since a PI first received NIH funding. We divide the estimate on funding by the mean employment in each category across all lab years.

Last, we study the demographic characteristics of the researchers who are touched by funding to explore representation in the research workforce and how it changes with funding. Figure 5A plots the gender composition of people working in a lab with the mean funding amount of $362,198 in 2018 BRDPI dollars (baseline) and one with $100,000 in additional funding. Gender is imputed using a modified version of Ethnea (*23*), an algorithm that predicts demographic characteristics using names. A limitation of this approach, in addition to the accuracy of the algorithm, is that it produces binary, nonfluid imputations of gender. The mean sized lab has slightly more women (3.89, SD = 8.60) than men (3.58, SD = 6.45), while gender is ambiguous for 1.78 (SD = 4.36) employees. A $100,000 increase in spending increases the employment of women by 0.26 percentage point (pp) (SE = 0.06) and men by 0.22 pp (SE = 0.04), which yields almost identical changes in percentage terms. Thus, larger projects do not seem to differ markedly from smaller projects in terms of their gender mix. The share of employees whose names are ambiguous declines slightly as projects grow. This appears to be a consequence of larger projects having a smaller share of Asian employees, for whom it is more difficult to impute gender.

Figure 5B reports estimates for four racial and ethnic groups [non-Hispanic Asians, Hispanics (of any race), non-Hispanic blacks, and non-Hispanic whites] imputed using Ethnicolr (https://pypi.org/project/ethnicolr/). The mean project has 6.88 (SD = 13.13) non-Hispanic whites compared to 1.67 (SD = 2.90) Asians and 0.3 to 0.4 Hispanics and non-Hispanic blacks. In percentage terms, the employment of non-Hispanic blacks followed by non-Hispanic whites increases the most as projects grow. Consequently, larger projects have lower shares of Asians and Hispanics. Figure S4 shows results with a richer ethnic classification based on Ethnea but without an explicit racial dimension. It shows considerable variation within the Asian category with the employment of Indians and Arabs responding considerably more positively to increases in funding than that of Chinese and other Asians.

Figure 5C reports estimates by age. The mean project employs 1.16 (SD = 3.26) people under age 25. The number of employees in each age bin declines monotonically from the 25-to-35 category until the 65+ category, with the mean project employing 1.60 (SD = 5.46) employees aged 65 and higher. In percentage terms, the employment of “prime” career workers (those 36 to 65 years old) responds the most to increases in funding (in percentage terms), with a peak for the 45 to 54 year old category. The employment of the youngest workers—under age 25—is, in percentage terms, as responsive as the 25 to 34 group, but the baseline employment of young workers is considerably lower. These estimates are broadly consistent with our occupation results (in Fig. 2), showing that the employment of faculty, research staff, other staff, and undergraduates is the most responsive to increases in funding.

Figure S5 shows demographic breakdowns for individual NIH ICs. Again, there are not only considerable similarities across ICs but also interesting differences. For instance, the NIA and National Institute of Child Health and Development tend to have a larger share of Western researchers and more women (and fewer researchers with uncertain gender predictions).

Overall, the amount of funding does not seem to greatly shift the demographic composition of projects. At the same time, there is some tendency for larger projects to be composed of older workers, more non-Hispanic blacks and non-Hispanic whites, and also slightly more women. Thus, the impact of funding on diversity is neither markedly different nor uniform across dimensions of representation.

## DISCUSSION

Funding stimulates research by supporting the teams that conduct it so that opening the black box of research projects requires accounting for how funding ripples through the people employed on research teams. We do this using unique, new data to study who is supported conducting research, what roles they play in labs, and how support from research projects ripples through to productivity. We find that large labs are more professionalized than smaller labs—with more faculty and staff. It seems plausible that this professionalization helps explain how large labs produce a high quantity of research without a diminution in the overall quality of research. This interpretation is consistent with our finding that larger and more research-active institutions and PIs with more experience are the driving force behind our results. We hypothesize that at these institutions, there are greater opportunities to professionalize labs as they grow.

While faculty—both PIs and other faculty—and their productivity are heavily affected by funding, so are other classes of employees, including trainees and staff. Our results show that the greatest effects of funding on research output are ripple effects on publications that do not include PIs. While funders often focus on research output from projects, they would be well advised to consider how funding ripples through the wide range of people employed in them.

If one thinks of science as being produced by a mix of labor, materials/capital, and knowledge, then the possible causes behind the increase in productivity from funding are an increase in these inputs. While we obtain similar estimates controlling for lab size, additional funding may well have benefits in terms of continuity of funding and attention devoted to research production (e.g., as opposed to proposal writing). We also show that the effects on purchased inputs are relatively modest. It is possible that expansions of labs generate higher productivity and ripple effects through knowledge spillovers and network effects. For instance, new people may bring new ideas and contacts into the lab that might influence the productivity of their colleagues in complex ways. Unfortunately, these effects are difficult to estimate because these three variables are determined simultaneously. To estimate these effects, one would need (quasi-) random variation in each of these inputs. We view identifying these variations and conducting such an analysis as an exciting avenue for future research.

## MATERIALS AND METHODS

### Data

#### UMETRICS and publication data

The UMETRICS data contain administrative information on all grant-related transactions at 72 public and private university campuses, which collectively account for 41% of federally funded academic R&D. While these institutions are not a random sample, they provide a valuable window into the academic research enterprise, especially at research intensive institutions. The data are drawn from transaction records on payments on research grants, making it possible to identify all the personnel, including trainees and staff, working on sponsored research projects, including people who are not included as authors on articles. The employee data have been annotated with imputed gender, race, and ethnicity (*24*) and include binned administrative data on birth years. We have self-reported, administrative data on gender for 12,867 faculty from one participating institution and, for that population, are able to impute gender for 95% of people with a precision of 93% overall, for men and for women. Using the same self-reported, administrative data on race and ethnicity, we are able to impute race and ethnicity for 60% of people with a precision of 90%. The employee data also include detailed job titles that have been classified into six categories: faculty, undergraduate students, graduate students, postdocs, research staff, and other staff, which includes all employees not classified elsewhere, who are themselves overwhelmingly staff (*25*). A more detailed breakdown is in table S2. Also included are transactions for purchased inputs, including equipment, services, and materials and supplies. Spending on subawards is calculated as the difference between total (direct) costs and spending on employees and purchase inputs.

The second core data asset is an update of the Author-ity disambiguation of the PubMed database (*14*, *15*). The dataset has been recently completely recomputed and updated to cover all the PubMed publications up to 2018. Torvik and Smalheiser probabilistically impute high-quality (98% accuracy) author clusters matched to funding records from NIH and National Science Foundation (NSF).

In this paper, we combine these two data assets first by matching PubMed to UMETRICS and then augmenting this match with the Author-ity publication clusters. Matching researchers to publications based on names is a challenging task. Here, we leverage the specific characteristics of PubMed, UMETRICS, and Author-ity data to overcome this issue. The matching procedure, which is sketched here, follows three steps. First, we assign each publication in PubMed to one or more projects in UMETRICS based on the grants acknowledged in the article. This importantly reduces the search space and consequently the computational time. We then try to associate each author on the article with researchers that have worked on at least one of the reported grants. Second, every researcher who was assigned at least one publication in the previous step is linked to her/his Author-ity cluster. Here, the matching is straightforward since the PubMed identification number (PMID)/authorship position pairs provide one-to-one links between the two datasets. Using Author-ity, we are able to attach UMETRICS researchers to all the articles they publish. Because Author-ity is generated independently of UMETRICS from publication data, it allows us to identify researchers’ publications regardless of whether or not (and for whatever reason) they acknowledge a UMETRICS project and to include publications that are outside of the UMETRICS coverage window, including those published before or after the researcher even works at a UMETRICS institution. Last, we iteratively extend the thus-obtained dataset by looking for coauthors that we might have missed in the first step taking advantage of the UMETRICS network of collaborators. More precisely, we take all the papers associated with a given researcher, and we search for his/her coauthors’ names among the people who collaborated with her/him on at least one UMETRICS project (not necessarily the ones acknowledged in the paper). Because the set of coauthors in Author-ity is small relative to the entire population of authors and the set of collaborators in UMETRICS is small relative to the entire population of employees, we have two well-defined populations with high probabilities of matching. The second and third steps are repeated until convergence.

#### PI and funding data

Once the publications are matched to authors in UMETRICS, we use a two-step procedure to combine these data with NIH ExPORTER’s comprehensive data on PIs and their funding. First, we identify unique PIs and all of their awards in NIH’s ExPORTER database. These are aggregated to generate funding at the level of PI years. During this process, we also generate a list of all transactions related to purchases, subawards, and personnel in UMETRICS who are paid on any of these NIH grants (regardless of whether they ever authored any publications). These too are aggregated to the level of PI years to generate a measure of inputs and lab composition. Second, we identify the specific people employed on any PI’s projects and link them to all their publications to the lab or PI with which they are associated. Descriptive statistics on the final sample of lab years are shown in Table 1.

**Table 1.** Lab year summary statistics. The table reports summary statistics (mean and SD) of the main variables used for the empirical analysis at the lab year level.

	**Mean**	**SD**
Unique publications authored by all employees	26.8	58.6
Unique research publications authored by all employees	23.3	49.8
Unique clinical publications authored by all employees	2.1	6.3
Total NIH funding	$362,198.1	$729,693.3
Mean Relative Citation Ratio (RCR) of publications	1.8	2.4
Highest RCR of publications	11.5	27.7
Total direct spending	$210,396.9	$433,997.1
Vendor spending	$41,495.4	$251,135.8
Subaward spending	$26,118.8	$150,416.1
Employee spending	$142782.7	$388,484.4
Total employees	9.3	17.0
Total Full Time Equivalents (FTEs)	1.9	4.6
Number of labs	10,202	
Number of lab years	78,701	

### Methods

#### Regression specifications

In Figs. 1, 2, and 5 and fig. S4, we estimate contemporaneous FEs regressions

$$Y_{\text{it}}=\alpha_{i}+\beta \cdot {\text{Funding}}_{\text{it}}+{\text{Year}}_{t}+{\text{CareerAge}}_{\text{it}}+\epsilon_{\text{it}}$$ (1)

where *Y_it_* is the outcome of interest, either lab spending on a particular category of expenses or the number of lab employees of a particular occupation or other category, and β is the coefficient of interest representing the association of lab funding with the given spending or employment category.

We also control for a full set of year dummies Year*_t_*, a full set of career age dummies CareerAge*_it_*, and the lab FE α*_i_*, which captures all effects of time-invariant lab characteristics. ϵ*_it_* is the residual error. We also cluster SEs at the lab level to account for correlated errors in the within-lab observations.

In our analysis of research outputs, we primarily use an event study design where the publication output of a lab is related to contemporaneous funding received as well as to leads and lags in funding. More concretely, the equation is given as

$$Y_{\text{it}}=\alpha_{i}+\sum_{n=-3}^{3}\beta_{n}{\text{Funding}}_{\text{it}+n}+{\text{Year}}_{t}+{\text{CareerAge}}_{\text{it}}+\epsilon_{\text{it}}$$ (2)

where *Y_it_* denotes publications authored by employees of lab *i* and published in year *t*, and Funding*_it_* is the amount of NIH funding received by this lab in that year. In other specifications, *Y* is publications authored by various subsets of the lab employees or the mean/maximum RCR of the publications by this lab. The control variables remain the same as in Eq. 1.

The event study is implemented by including three past values (*n* < 0) and three future values (*n* > 0) of this variable in addition to the contemporaneous one (*n* = 0). The coefficients β*_n_*, which we plot in Figs. 3 and 4 and fig. S2 (fig. S3 plots the sum, β_1_ + β_2_ + β_3_ for NIH ICs), represent the association of funding in year (*t* + *n*) with publications in year *t*.
