# Supplementary material for: The ripple effects of funding on researchers and output
Source: Sci Adv. 2022 Apr 22;8(16):eabb7348. doi: 10.1126/sciadv.abb7348 (PMC9032967; doi:10.1126/sciadv.abb7348)
Supplement: Supplementary file 1 — Tables S1 and S2 Figs. S1 to S5 [file sciadv.abb7348_sm.pdf]

Science Advances
AAAS

Supplementary Materials for

The ripple effects of funding on researchers and output

Reza Sattari, Jung Bae, Enrico Berkes, Bruce A. Weinberg*

*Corresponding author. Email: weinberg.27@osu.edu

Published 22 April 2022, *Sci. Adv.* **8**, eabb7348 (2022)
DOI: 10.1126/sciadv.abb7348

This PDF file includes:

Tables S1 and S2
Figs. S1 to S5

Table S.1 Breakdown of occupations

Occupation	Headcount	Share
Faculty	41,052	12.42
Postdoc	31,079	9.40
Graduate Student	70,077	21.20
Research Staff		
Research	42,265	12.79
Research Facilitation	56,062	16.96
Other Staff		
Clinical	5,552	1.68
Instructional	4,942	1.5
Other	17,814	5.39
Technical Support	6,054	1.83
Undergraduate	55,581	16.82
Total	330,478	100

Note: The table reports the number and share of unique people by occupation before aggregating them into broader categories. In our analysis, we combine Research and Research Facilitation into "Research Staff" and Clinical, Instructional, Other (who manual inspection indicates are overwhelmingly staff), and Technical into "Other Staff". The numbers reflect the distribution based on the initial occupation reported in UMETRICS for each person.

Table S.2 Publication timing relative to appearance in lab

Occupation	3+ years before	Within 3 years	During	Within 3 years after	3+ years after
Faculty	111,630	261,896	901,029	348,637	265,280
Graduate Student	4,136	14,534	37,903	44,527	41,106
Other Staff	3,899	9,891	23,280	14,104	8,985
Postdoc	8,135	26,262	55,623	54,352	46,100
Research Staff	20,170	50,152	135,346	87,568	71,681
Undergraduate	231	1,409	3,626	5,477	4,307
Total	148,201	364,144	1,156,807	554,665	437,459

Note: The table reports distribution of papers by occupation and timing of publication relative to the employment in a certain lab. Particularly, we report the number of papers that were published

before a researcher appears paid by a certain lab for the first time in our sample, after the last transaction in our records, and between the two. The unit of observation is a lab- or PI-year.

Figure S.1. Breakdown of Spending and Employment by NIH Institutes and Centers

A. Spending

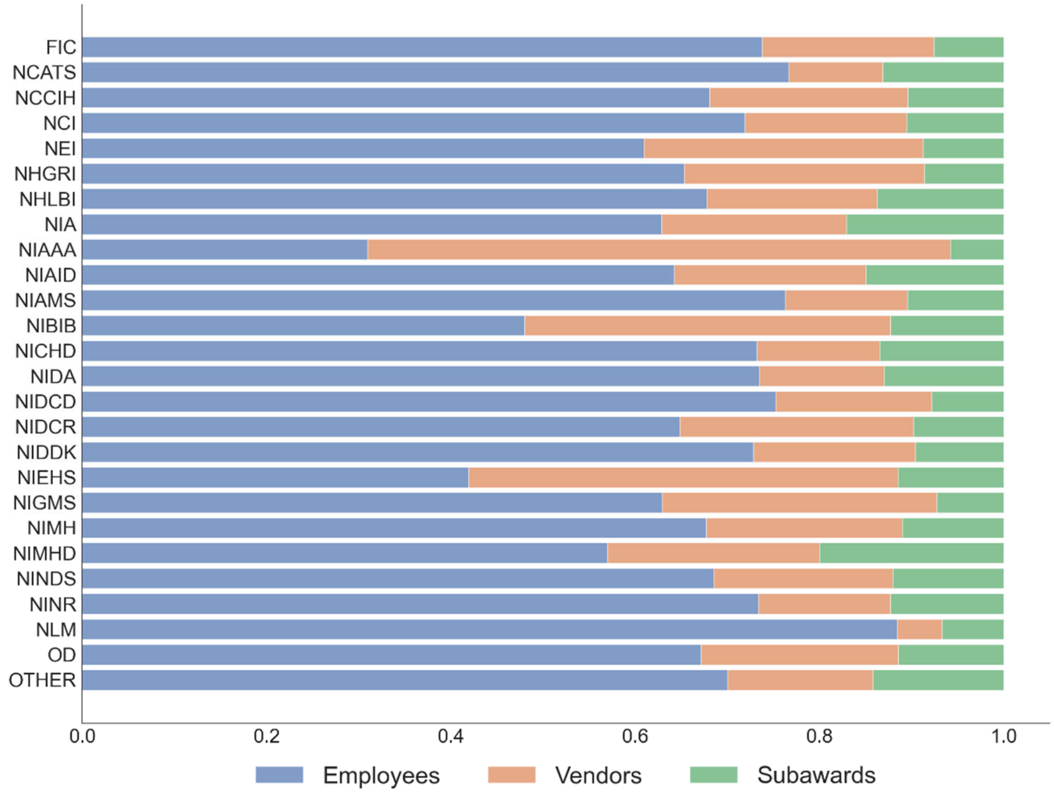

Note: The figure shows the breakdown of spending for individual NIH Institutes and Centers (ICs).

B. Occupation

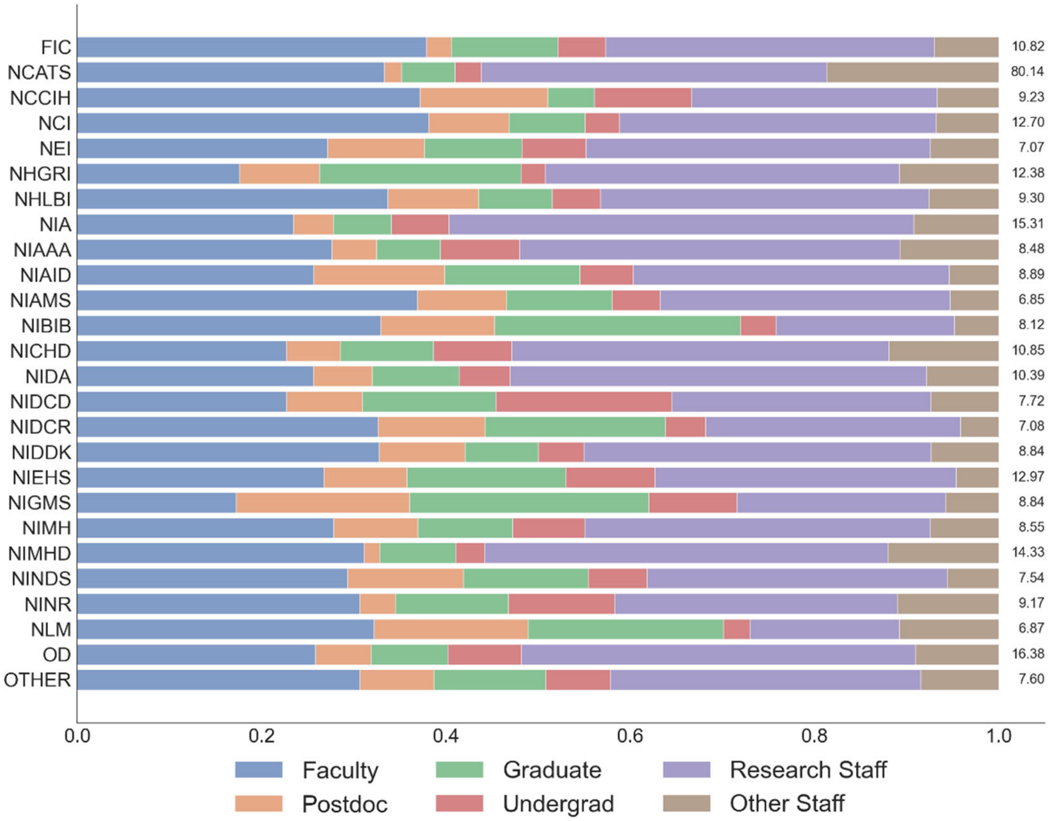

Note: The figure shows the breakdown of employment by occupation category for individual NIH Institutes and Centers (ICs). The numbers at the right show the total number of employees per award for each IC. Employment is measured as full-time equivalent workers and is estimated by prorating people by the share of their time charged to each project, their full-time / part-time status, and the number of days worked during the year.

Figure S.2.A. Ordinary Least Squares (OLS) versus Fixed Effects (FE) Estimates

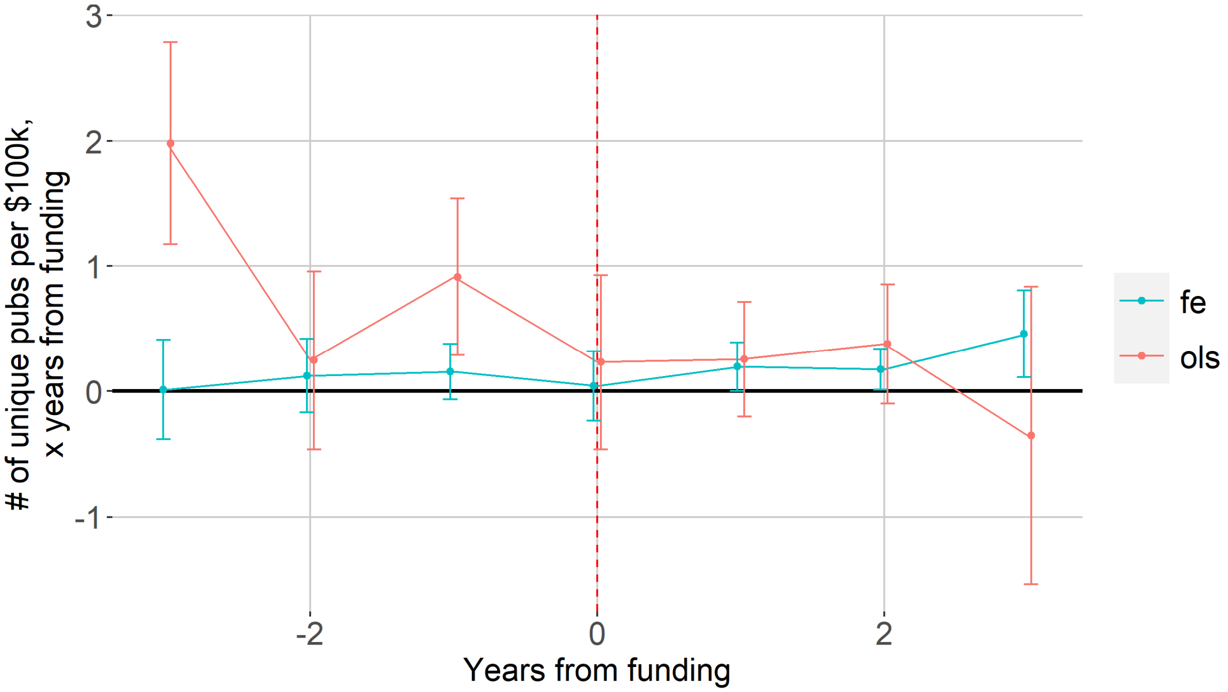

Note: The figure plots the relationship between funding to a lab (measured in 2018 BRDPI dollars) and unique publications authored by the people in that lab, from publications 3 years before funding is received (-3) to 3 years after funding was received (+3). It reports estimates for all unique publications using ordinary least squares (OLS) and lab or PI fixed effects (FE) models. The FE models account for all time-invariant differences across labs. Both estimates control for calendar year fixed effects and for the number of years since a PI first received NIH funding. Ninety-five percent confidence intervals are shown in all panels. Standard errors are clustered at the lab level.

Figure S.2.B. Publications controlling for lab size

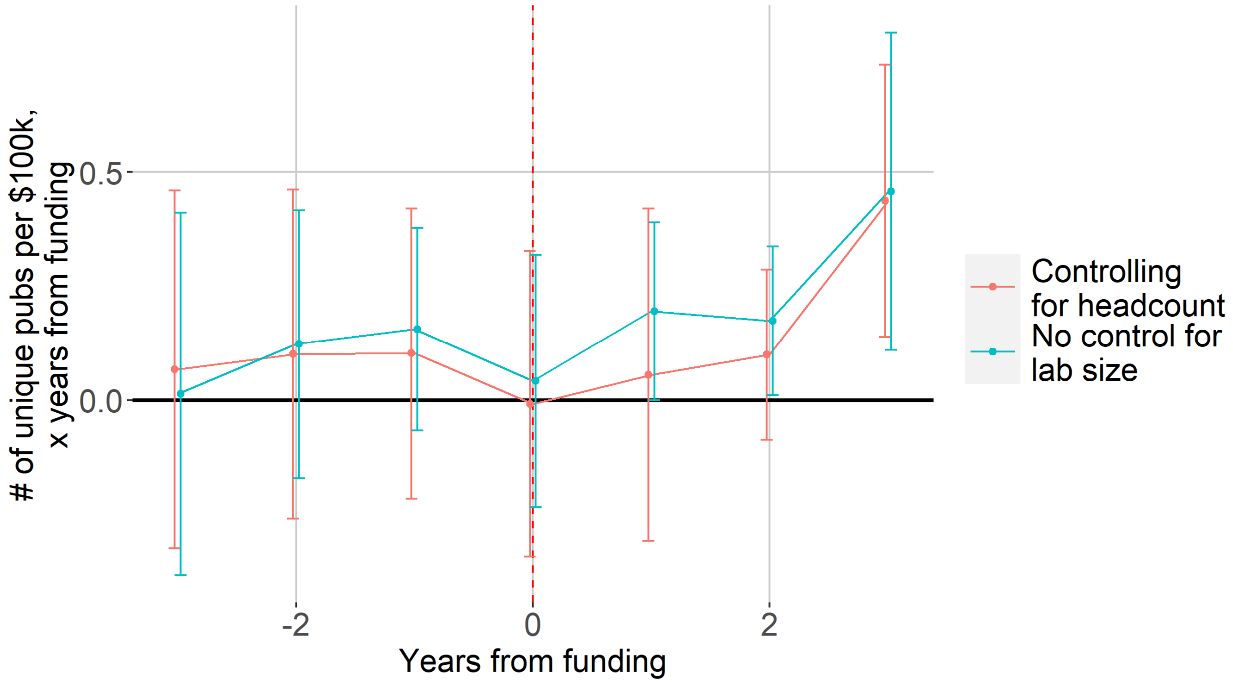

Note: The figure plots the relationship between funding (measured in 2018 BRDPI dollars) to a lab and unique publications authored by the people in that lab, from publications 3 years before funding is received (-3) to 3 years after funding was received (+3). This figure plots estimates with controls for the number of employees in the lab (red series) and without this control (turquoise series). Both series control for lab or PI fixed effects, calendar year fixed effects, and for the number of years since a PI first received NIH funding. Ninety-five percent confidence intervals are shown in all panels. Standard errors are clustered at the lab level.

Figure S.2.C. Research and clinical publications

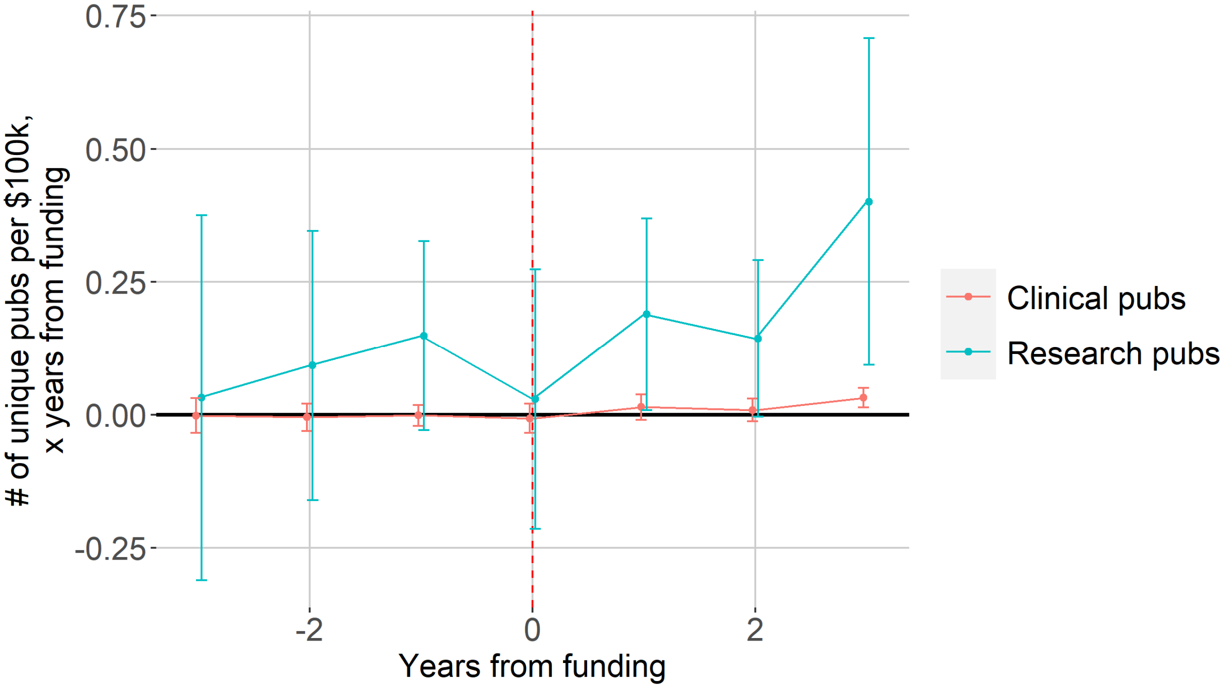

Note: The figure plots the relationship between funding to a lab (measured in 2018 BRDPI dollars) and unique publications authored by the people in that lab, from publications 3 years before funding is received (-3) to 3 years after funding was received (+3). These series restrict the outcome variable to clinical publications (red series), defined as clinical trials, systematic reviews, and clinical guidelines, and research publications (turquoise series) as defined by the iCite database from the National Institutes of Health, Office of Portfolio Analysis. Both sets of estimates control for lab or PI fixed effects, calendar year fixed effects, and for the number of years since a PI first received NIH funding. Ninety-five percent confidence intervals are shown in all panels. Standard errors are clustered at the lab level.

Figure S.2.D. Mean and PI-max RCR of publications

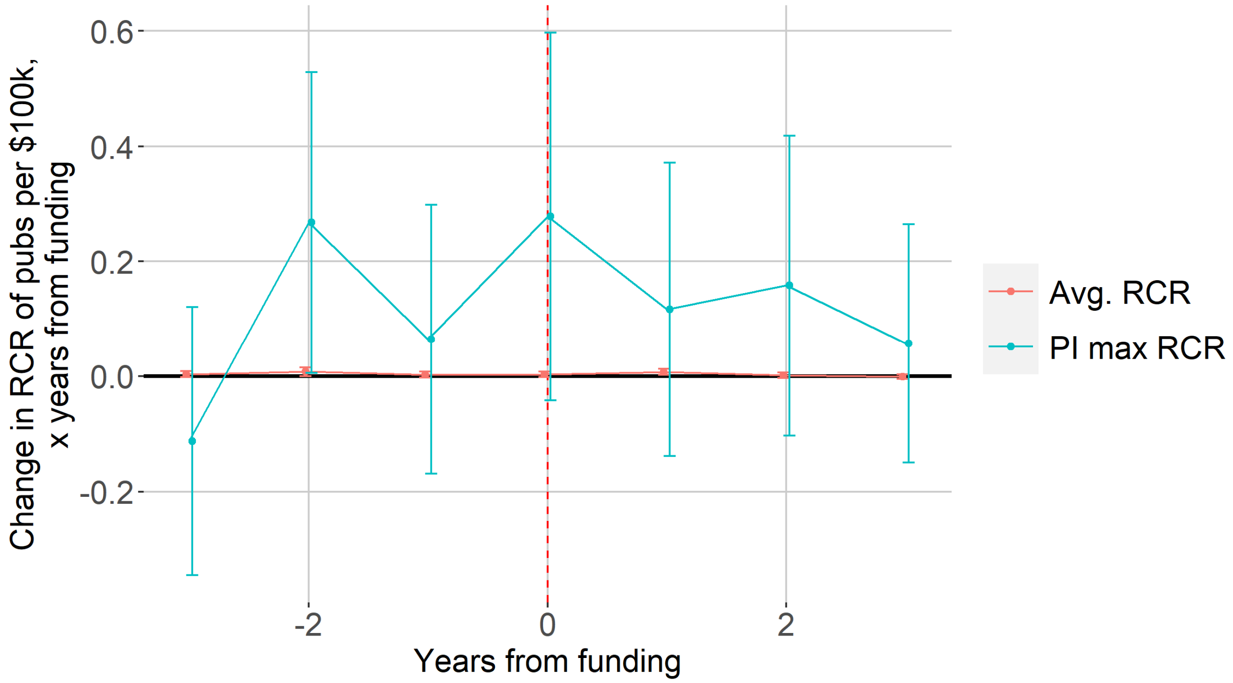

Note: The figure plots the relationship between funding to a lab (measured in 2018 BRDPI dollars) and the quality measures of publications authored by the people in that lab, from publications 3 years before funding is received (-3) to 3 years after funding was received (+3). The quality measure is the relative citation ratio (RCR): the article's mean citations per year divided by the median number of citations received by papers in the same research area published at the same time. We plot effects on the mean RCR of all papers published by the lab (red series) and the maximum RCR of those papers (turquoise series). Both estimates control for lab or PI fixed effects, calendar year fixed effects, and for the number of years since a PI first received NIH funding. Ninety-five percent confidence intervals are shown in all panels. Standard errors are clustered at the lab level.

Figure S.2.E. Publications by Type of Award.

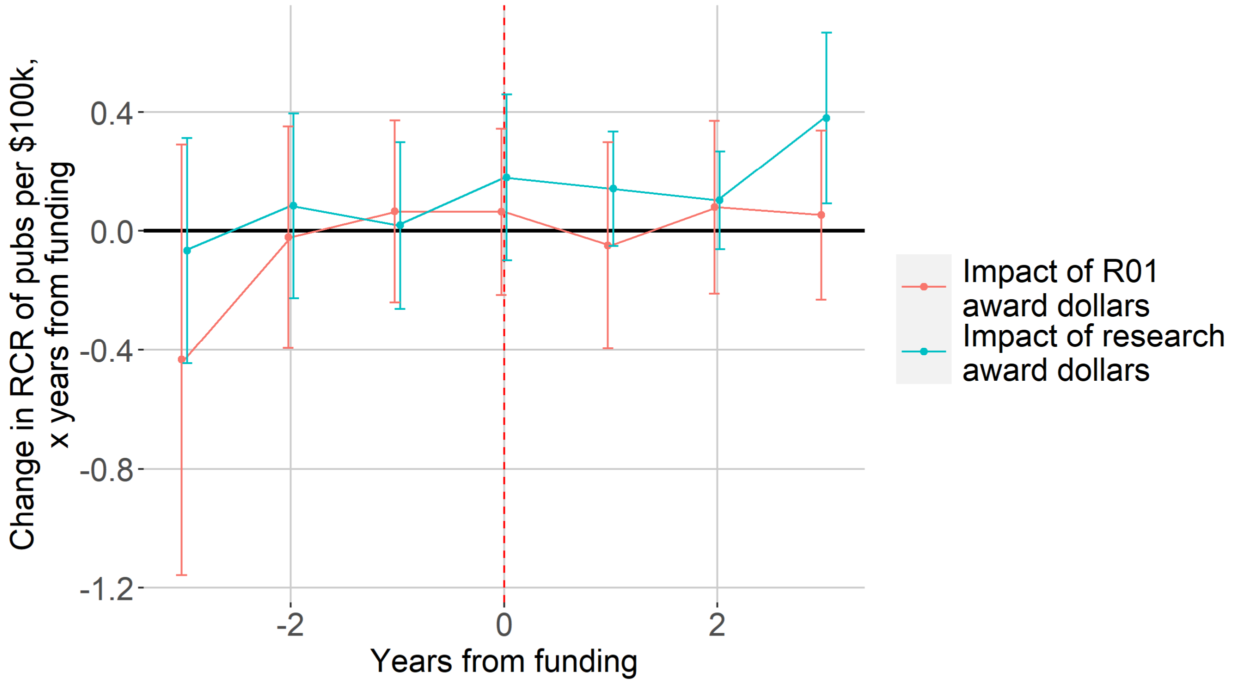

Note: The figure plots the relationship between funding to a lab (measured in 2018 BRDPI dollars) and unique publications authored by the people in that lab, from publications 3 years before funding is received (-3) to 3 years after funding was received (+3). This figure restricts the measure of funding to money received through the R01 award mechanism (red series) and through research grants (all R-class mechanisms including R01, plus K99 and U01, turquoise series). Both estimates control for lab or PI fixed effects, calendar year fixed effects, and for the number of years since a PI first received NIH funding. Ninety-five percent confidence intervals are shown in all panels. Standard errors are clustered at the lab level.

Figure S.2.F. Institutions by Number of Doctoral Degrees Awarded

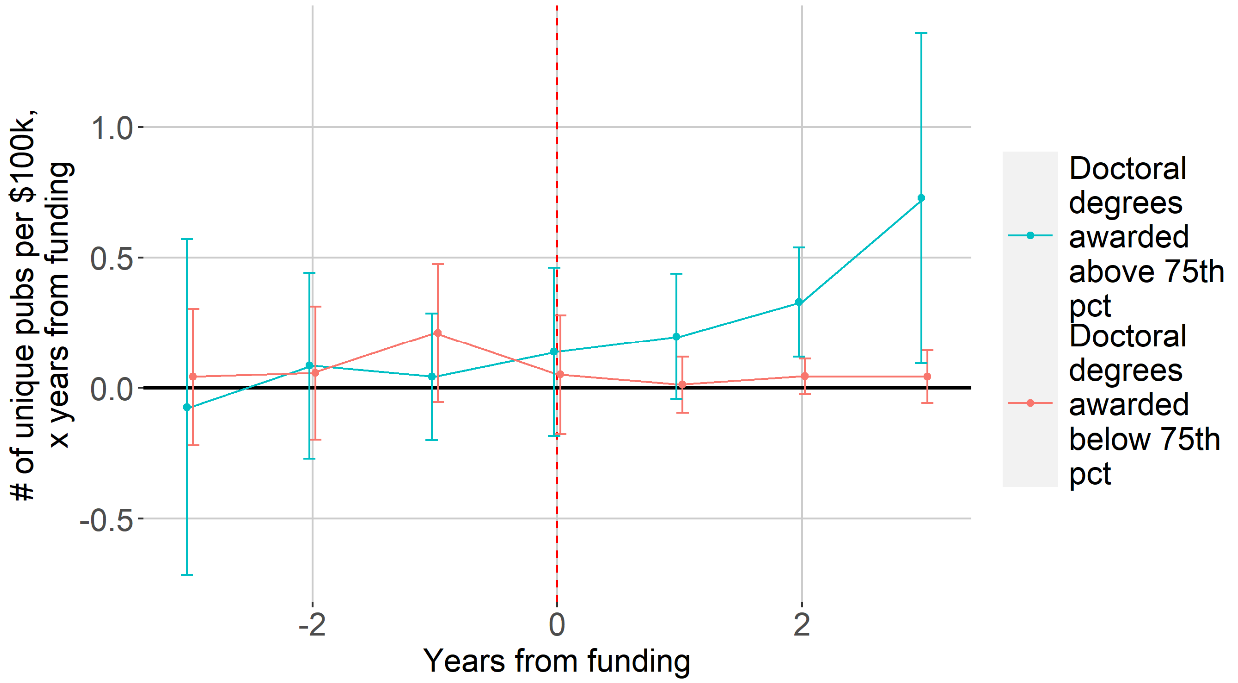

Note: The figure plots the relationship between funding to a lab (measured in 2018 BRDPI dollars) and unique publications authored by the people in that lab, from publications 3 years before funding is received (-3) to 3 years after funding was received (+3). To perform these regressions, we split our sample into institutions in the bottom 75th percentile in terms of average annual number of doctoral degrees awarded (red series), and in the top 25th percentile (turquoise series). Both sets of estimates control for lab or PI fixed effects, calendar year fixed effects, and for the number of years since a PI first received NIH funding. Ninety-five percent confidence intervals are shown in all panels. Standard errors are clustered at the lab level.

Figure S.2.G. Institutions by Enrollment

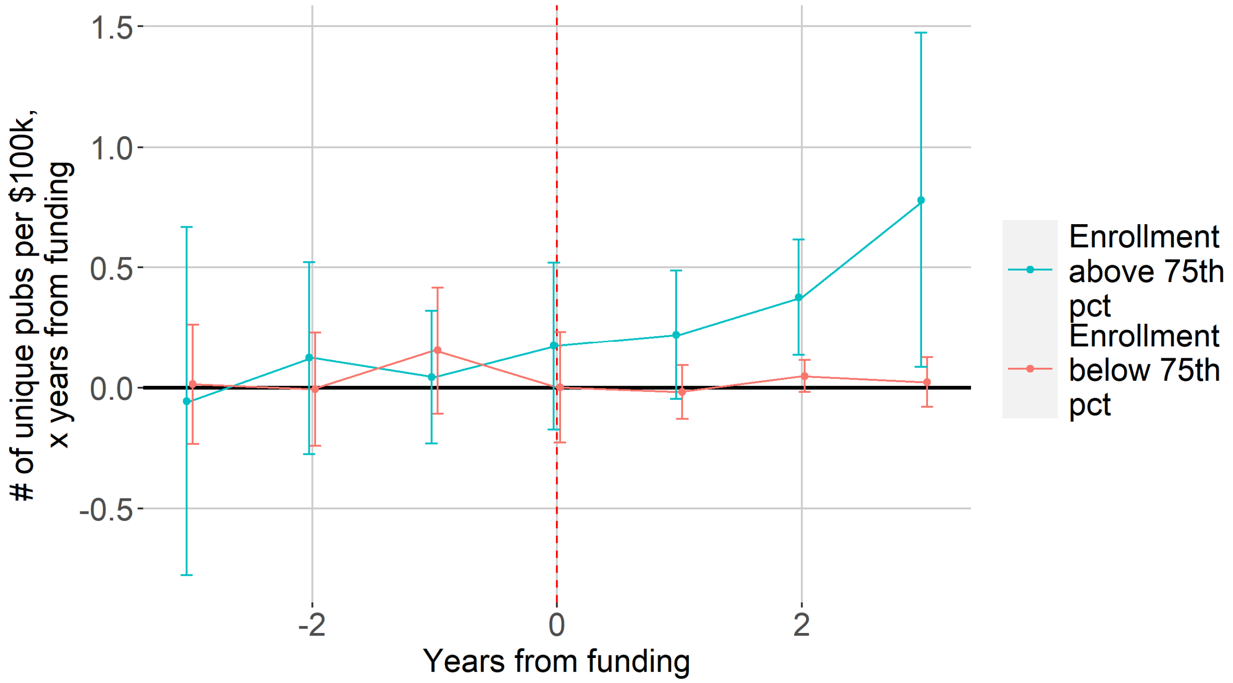

Note: The figure plots the relationship between funding to a lab (measured in 2018 BRDPI dollars) and unique publications authored by the people in that lab, from publications 3 years before funding is received (-3) to 3 years after funding was received (+3). To perform these regressions, we split our sample into institutions in the bottom 75th percentile in terms of average annual enrollment (red series), and in the top 25th percentile (turquoise series). Both sets of estimates control for lab or PI fixed effects, calendar year fixed effects, and for the number of years since a PI first received NIH funding. Ninety-five percent confidence intervals are shown in all panels. Standard errors are clustered at the lab level.

Figure S.2.H. Institutions by Federal Funding

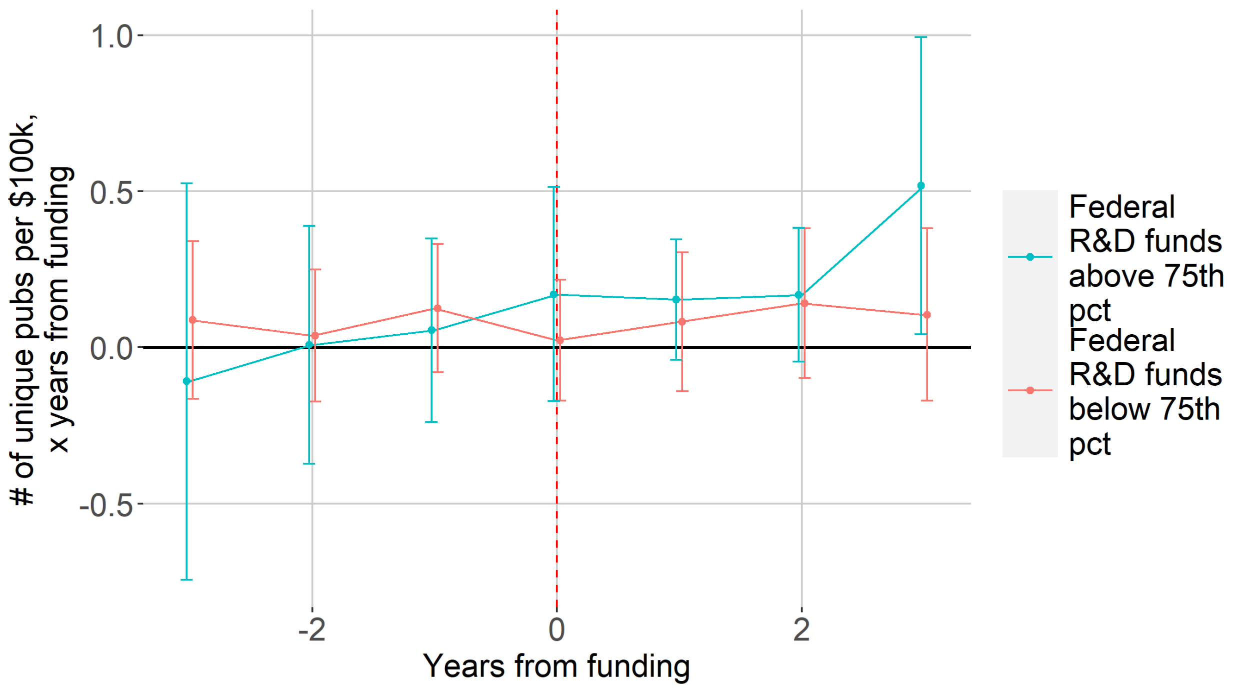

Note: The figure plots the relationship between funding to a lab (measured in 2018 BRDPI dollars) and unique publications authored by the people in that lab, from publications 3 years before funding is received (-3) to 3 years after funding was received (+3). To perform these regressions, we split our sample into institutions in the bottom 75th percentile in terms of average annual federal funding (red series), and in the top 25th percentile (turquoise series). Both sets of estimates control for lab or PI fixed effects, calendar year fixed effects, and for the number of years since a PI first received NIH funding. Ninety-five percent confidence intervals are shown in all panels. Standard errors are clustered at the lab level.

Figure S.2.I. Career Age

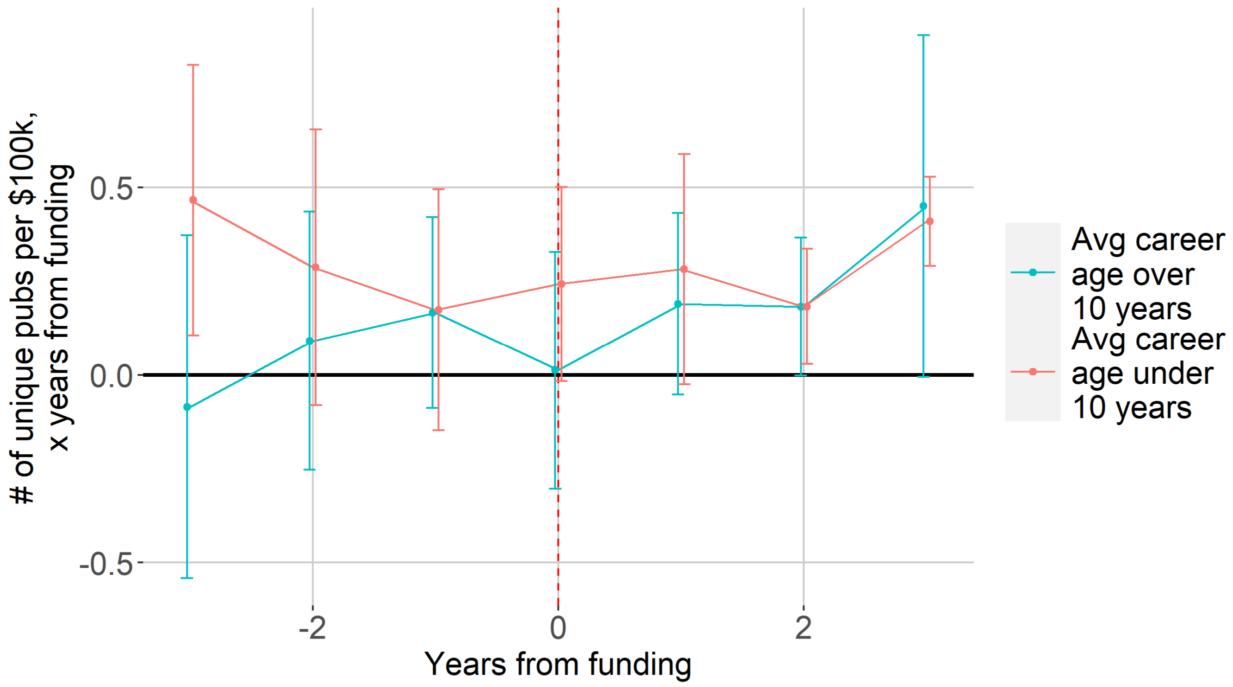

Note: The figure plots the relationship between funding to a lab (measured in 2018 BRDPI dollars) and unique publications authored by the people in that lab, from publications 3 years before funding is received (-3) to 3 years after funding was received (+3). To perform these regressions we split our sample into PIs with an average career age below 10 years (red series), and above 10 years (turquoise series). Both sets of estimates control for lab or PI fixed effects, calendar year fixed effects, and for the number of years since a PI first received NIH funding. Ninety-five percent confidence intervals are shown in all panels. Standard errors are clustered at the lab level.

Figure S.3. Funding Effects by NIH IC

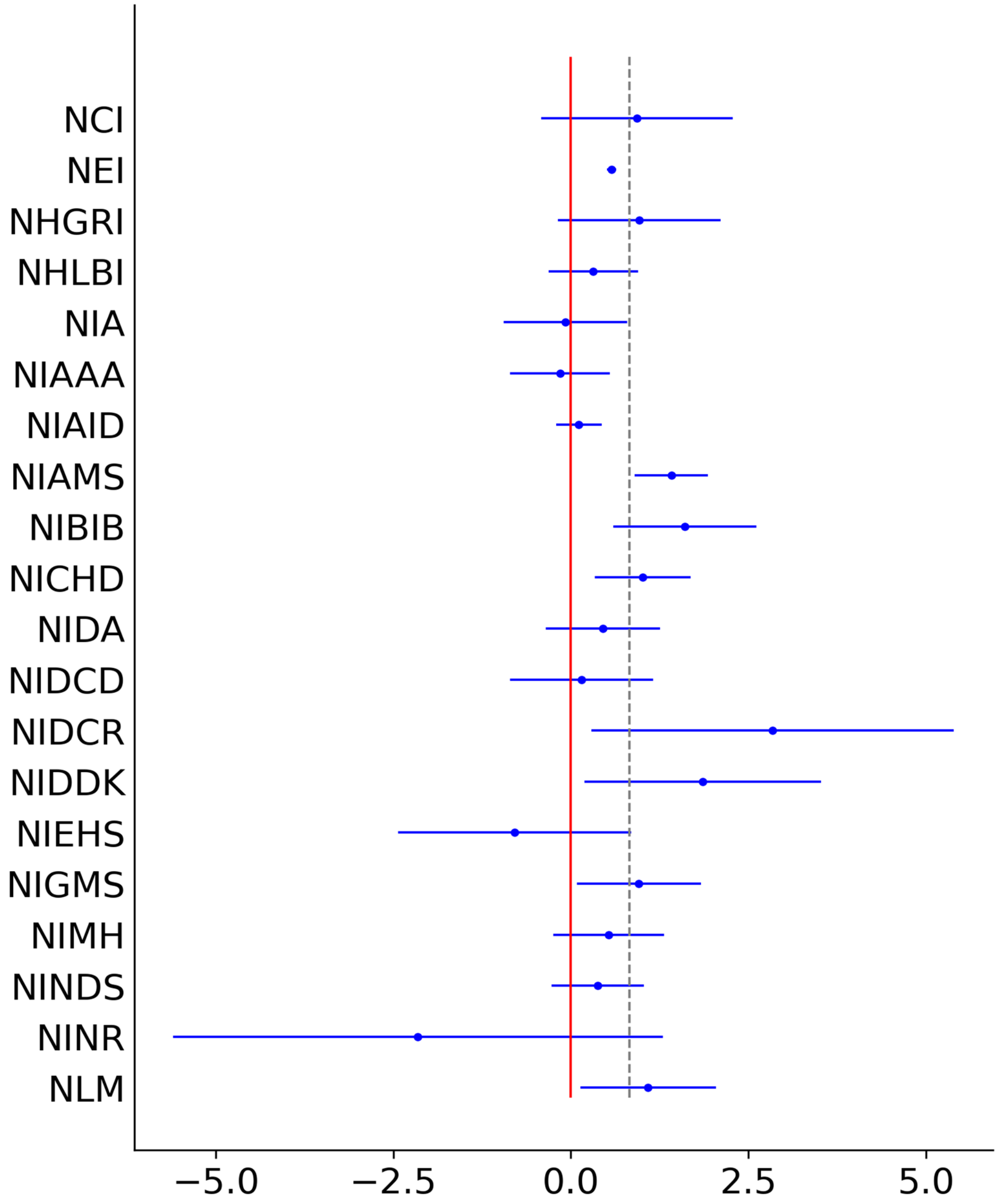

Note: The figure summarizes the relationship between funding to a lab (measured in 2018 BRDPI dollars) and the number of unique publications authored by the people in that lab over the 3 years following funding, separately for each NIH Institute and Center (IC) for which we have sufficient data. Each plotted coefficient is the sum of the coefficients on funding received one, two, and three years prior to a focal publication year. All estimates control for lab or PI fixed effects, calendar year fixed effects, and for the number of years since a PI first received NIH funding. Ninety-five percent confidence intervals are calculated as described in Appendix B. Standard errors are clustered at the lab level.

Figure S.4. Ethnic Composition of Projects

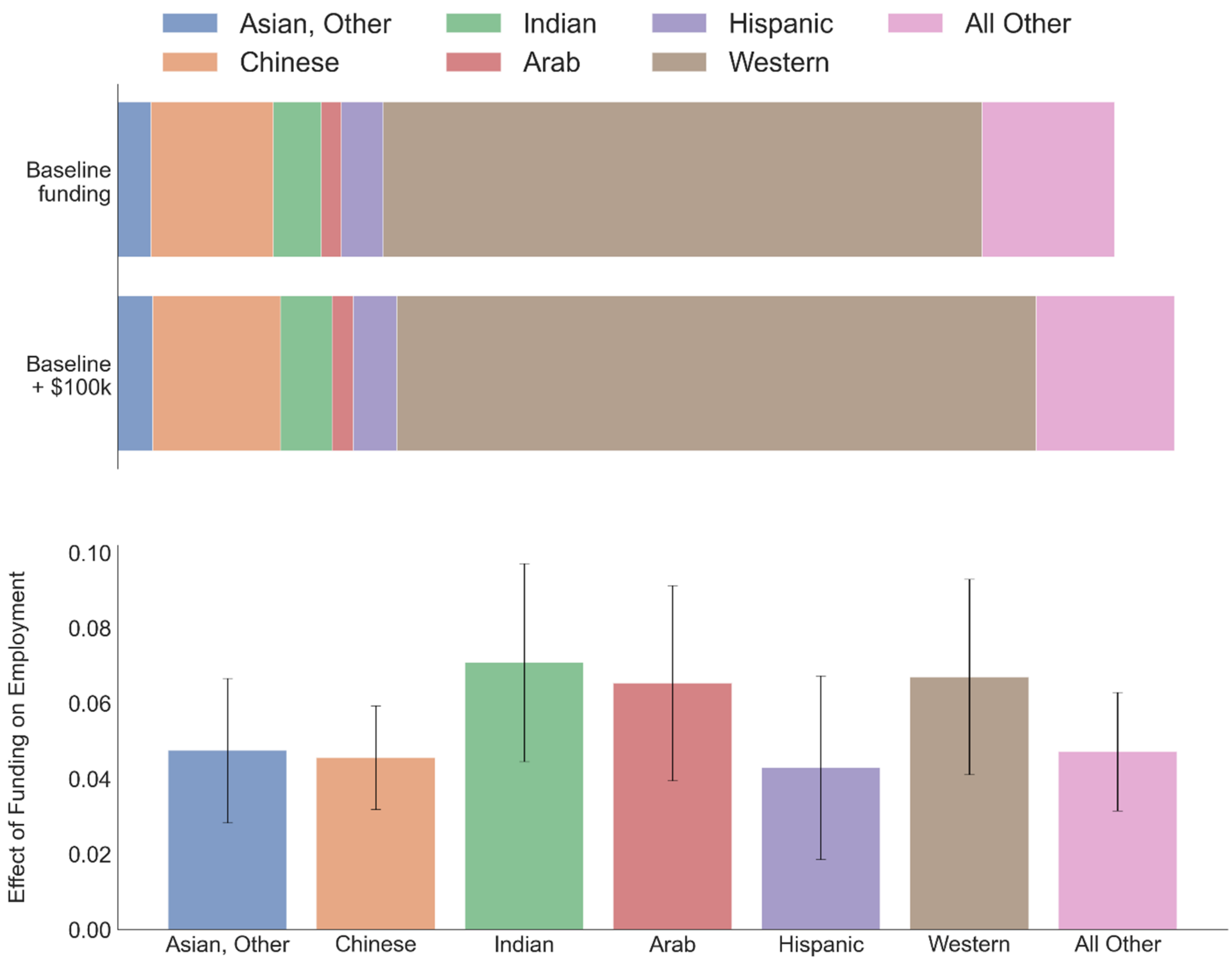

Note: The top panel shows how (direct) spending is related to the employment measured in full time equivalent workers for employees of different ethnicities for a lab of the mean size of $362,198 in 2018 BRDPI dollars (baseline) and a lab with $100,000 in additional spending. Full time equivalent workers are estimated by prorating people by the share of their time charged to each project, their full-time / part-time status, and the number of days worked during the year. The bottom portion shows the percentage change in full time equivalent employment of each type of employee from a $100k increase in funding (along with 95% confidence intervals). Standard errors are clustered at the lab level. These are estimated from a regression of employment of each type of employee on total funding for that lab in each year controlling for lab or PI fixed effects, calendar year fixed effects, and for the number of years since a PI first received NIH funding. We divide the estimate on funding by the mean spending in each category across all lab-years. "Asian, Other" includes Indonesian, Japanese, Korean, Thai, and Vietnamese; "Western" includes Baltic, Dutch, English, French, German, Greek, Hungarian, Israeli, Italian, Nordic, Romanian, Slav, and Turkish; "All Other" includes African, Caribbean, and Unknown.

Figure S.5. Breakdown of Employment by NIH Institutes and Centers.

A. Gender

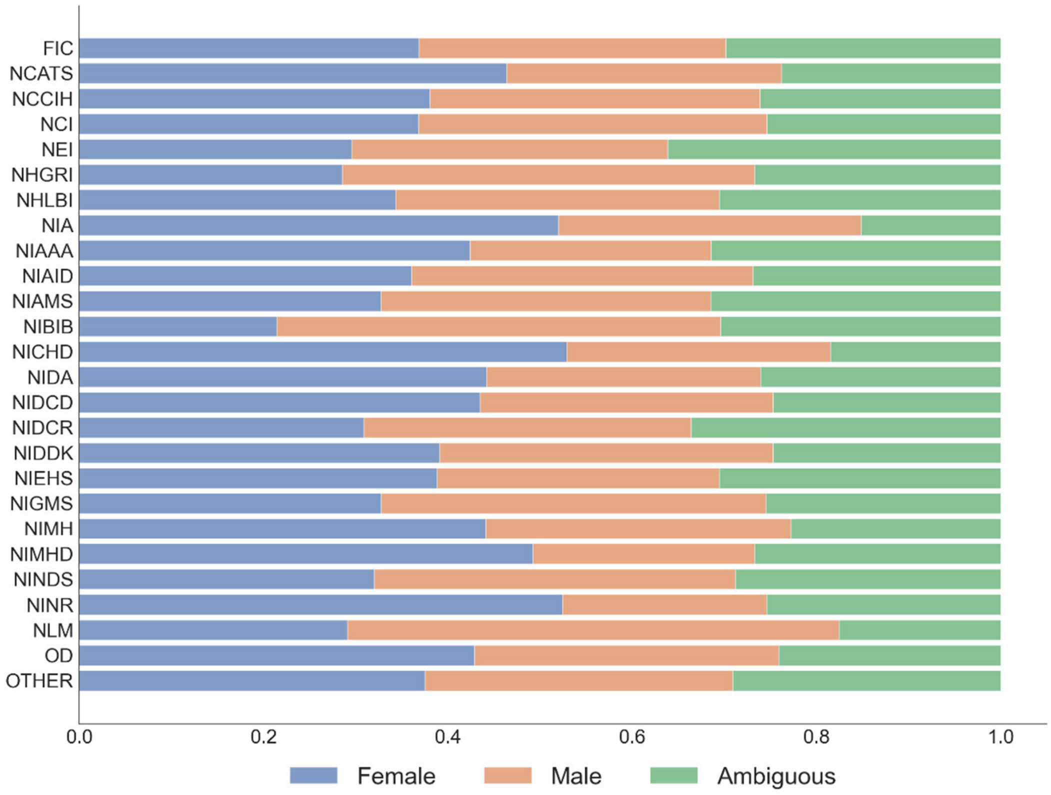

Note: The figure shows the breakdown of employment by imputed gender for individual NIH Institutes and Centers (ICs). Employment is measured in annual full-time equivalents by prorating people by the share of their time charged to each project, their full-time / part-time status, and the number of days worked during the year.

B. Race and Ethnicity

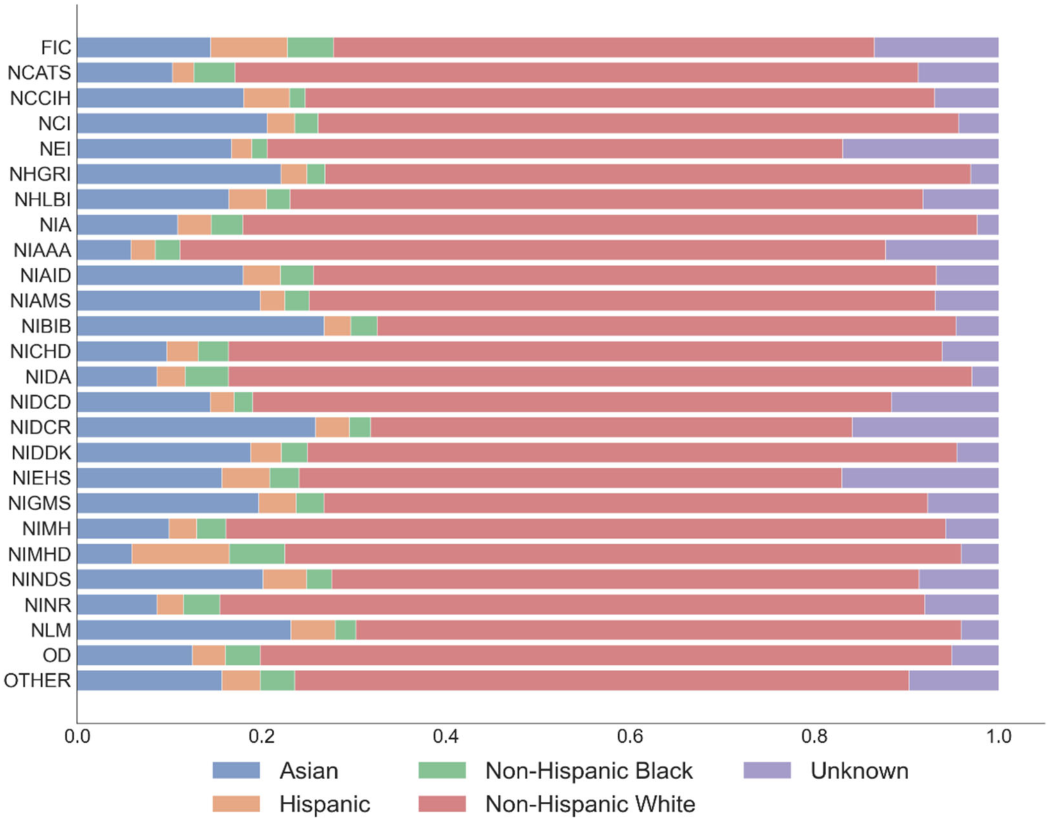

Note: The figure shows the breakdown of employment by imputed race for individual NIH Institutes and Centers (ICs). Employment is measured in annual full-time equivalents by prorating people by the share of their time charged to each project, their full-time / part-time status, and the number of days worked during the year.

C. Ethnicity

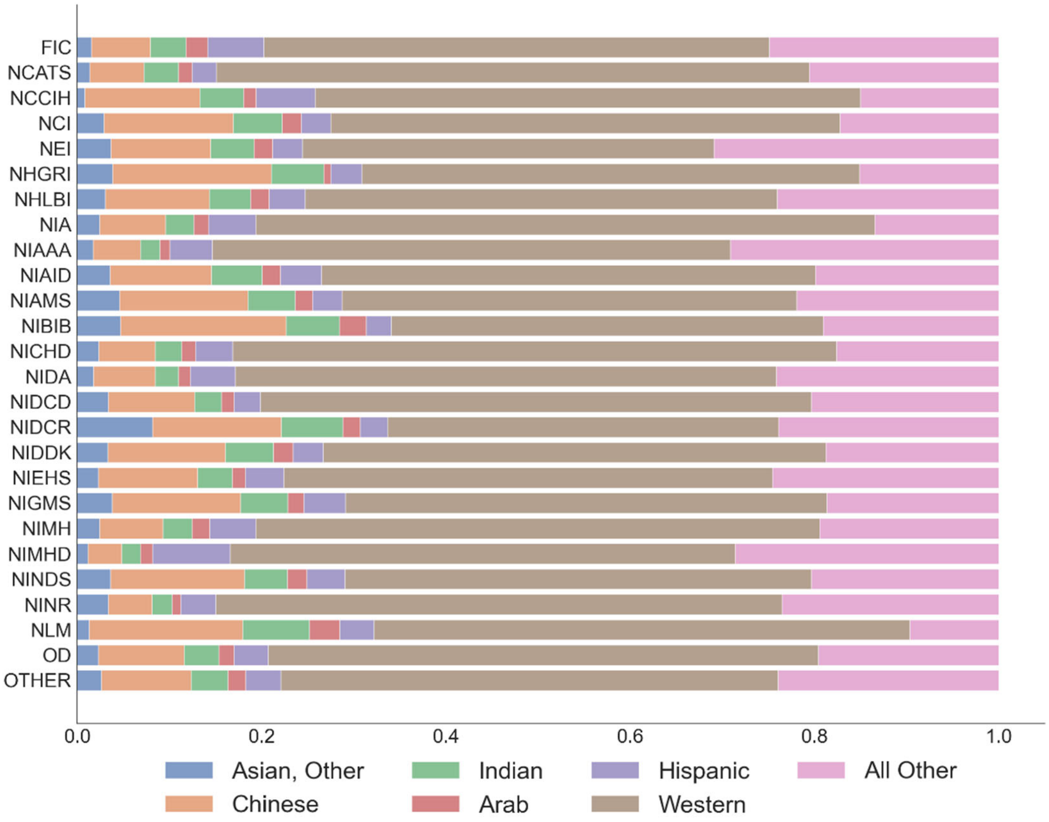

Note: The figure shows the breakdown of employment by imputed ethnicity for individual NIH Institutes and Centers (ICs). "Asian, Other" includes Indonesian, Japanese, Korean, Thai, and Vietnamese; "Western" includes Baltic, Dutch, English, French, German, Greek, Hungarian, Israeli, Italian, Nordic, Romanian, Slav, and Turkish; "All Other" includes African, Caribbean, and Unknown. Employment is measured in annual full-time equivalents by prorating people by the share of their time charged to each project, their full-time / part-time status, and the number of days worked during the year.

D. Age

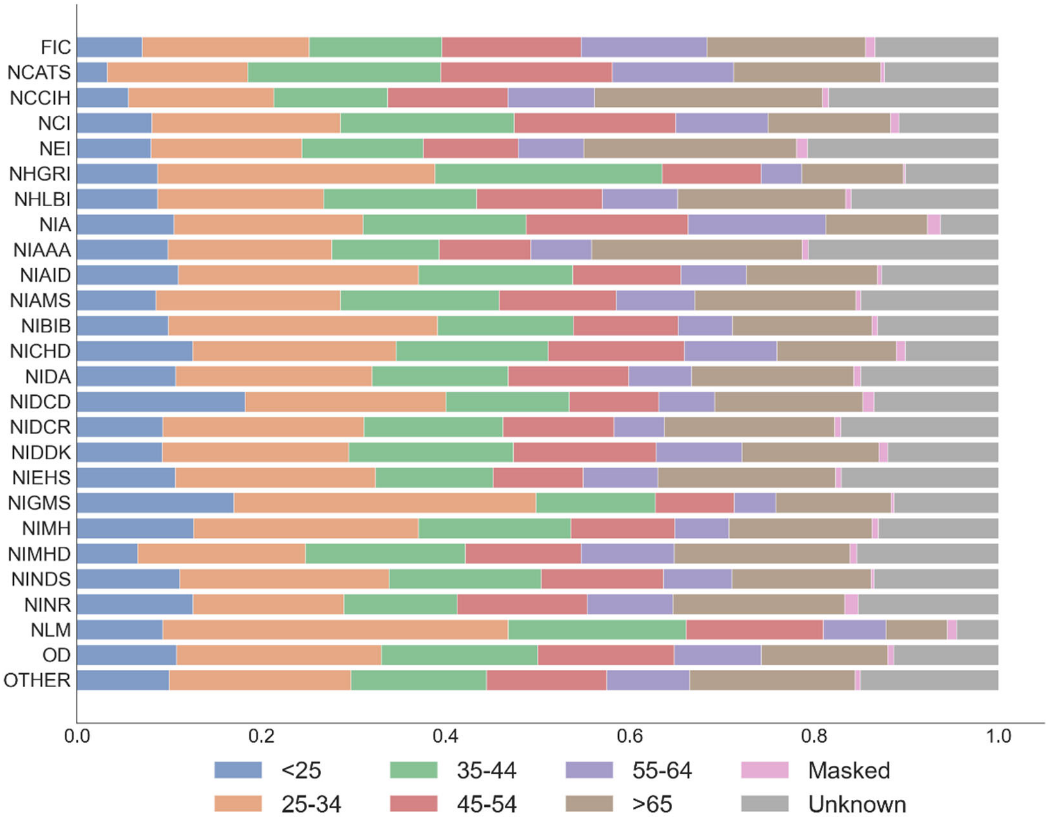

Note: The figure shows the breakdown of employment by age for individual NIH Institutes and Centers (ICs). Employment is measured in annual full-time equivalents by prorating people by the share of their time charged to each project, their full-time / part-time status, and the number of days worked during the year.
